# Reviewed article: Souza MA, Lopes LC, Silva MT. Factors associated with complications related to the use of peripherally inserted central venous catheter in hospitalized children:a case-control study, Sorocaba, Brazil, 2018-2022. Epidemol Serv Saude. 2025:34;e20240134

**DOI:** 10.1590/S2237-96222025v34e20240134.b

**Published:** 2025-09-08

**Authors:** Maria Auxiliadora Parreiras Martins

**Affiliations:** 1 Universidade Federal de Minas Gerais, Belo Horizonte, MG Brazil Universidade Federal de Minas Gerais Belo Horizonte MG Brazil

## First round comments

Prezados autores, agradecemos o envio da resposta aos comentários dos revisores. Após verificação do manuscrito, observou-se que as modificações não foram atendidas de modo satisfatório. Ainda há dificuldade de compreensão de conceitos, métodos empregados e resultados apresentados. Importante realizar nova revisão no texto utilizando o guia de redação de estudos caso-controle para refinamento do texto https://www.equator-network.org/wp-content/uploads/2015/10/STROBE_checklist_v4_case-control.pdf Recomendo a observância aos comentários do editor para que sejam realizados os ajustes solicitados. Esclareço que se as recomendações detalhadas a seguir não forem atendidas integralmente o manuscrito não poderá ser aceito para publicação.

1- Houve dúvida quanto emprego de amostra que foi definido a posteriori, quando os dados já estavam coletados. Foi explicado no texto que a decisão foi à “frequência maior no uso em pacientes pediátricos na primeira infância”. Importante que o cálculo amostral seja feito antes da coleta de dados. No caso de emprego de amostra de conveniência, pode-se calcular o poder estatístico alcançado após as análises. Reconhecer esse aspecto como limitação do estudo no texto.

2- Outro aspecto é que foi usada expressão ampla “primeira infância” que deve ser removida do texto e substituída pela faixa etária específica (até 2 anos 11 meses e 29 dias de idade). Conceito de “Primeira Infância”: o Ministério da Saúde conceitua como “período que abrange os primeiros 6 (seis) anos completos ou 72 (setenta e dois) meses de vida da criança”. Porém, o estudo não inclui todas crianças na primeira infância. Além disso, seria importante justificar o porquê da exclusão das 13 crianças de faixa etária entre 3 e 6 anos.

3- Ajustar o título de acordo com o objetivo e conteúdo apresentado. Sugiro alterar a informação “complicações durante o uso de Cateter Venoso Central de Inserção Periférica” para “complicações relacionadas ao uso de Cateter Venoso Central de Inserção Periférica”. Além disso, remover menção à “primeira infância”.

4- Importante descrever de forma clara o desfecho de interesse no estudo, no caso as complicações relacionadas ao uso do Peripherally Inserted Central Catheter (PICC)? Nos métodos estatísticos foi relatada investigação da associação entre as complicações mais frequentes e a obstrução com o PICC. Porém, na seção dos resultados entende-se que o resultado de interesse não era apenas obstrução, mas também a ocorrência de outras complicações decorrentes do uso do PICC (ver tabela 3). Indicar claramente os critérios ou métodos utilizados para a determinação dessas complicações.

5- Na Figura 1, o critério de exclusão “Passagem de CVC (n= 194)” não foi mencionado nos métodos. Além disso, os pacientes controles foram divididos em alta hospitalar (n= 28), desvio de qualidade (n= 9) e contrarreferência com PICC (n=10). Esses critérios não foram descritos nos métodos.

6- O cálculo das frequências relativas nas tabelas está extremamente confuso quanto ao n total utilizado para cálculo. Na tabela 2, foi acrescentado o valor “n=62” ao título. Entendo que esse valor deveria ser considerado como 100% para realização dos cálculos. Porém, foi considerado como 100% a amostra total do estudo (n=109). Na tabela 4, foi considerado como 100% o valor de cada variável na amostra total (n=109). 

No entanto, o valor n representado na tabela é igual a 33. Não entendi, por exemplo, as variáveis “tipo de cirurgia” e “comorbidades” na tabela 4. Lembrar que as tabelas devem auto-explicativas.

7- A magnitude do problema estudado e o impacto das complicações relacionadas ao uso do PICC para os pacientes investigados e para os sistemas de saúde não foi discutido com profundidade.

8- Página 3 (linhas 8-9): “No Brasil, poucos estudos avaliaram sua utilização em sistemas universais de saúde em pacientes cirúrgicos pediátricos na primeira infância.” Recomenda-se fazer menção ao Sistema Único de Saúde de forma clara e direta.

9- Na página 7 (linhas 53-59): “Não foram encontrados na literatura estudos que abordassem a associação da Síndrome de Down com o fator de obstrução em cateteres de inserção periférica ou central. Entre as quatro crianças com síndrome de Down, três eram cardiopatas.”

Penso que aqui há um importante fator de confusão. Pacientes cardiopatas tem maiores chances de complicação em cateterismos? Considerando que sim (https://doi.org/10.1590/S2179-83972007000100010), o fator relacionado a maiores complicações no cateterismo é a síndrome de Down ou as prevalentes alterações cardiovasculares nesses indivíduos? Os autores citam que “na literatura nacional e internacional, o sexo é abordado de forma mais ampla, sem associação direta ao fator de obstrução ou complicação (14, 19)”, porém os estudos indicados na referência não abordam dados nacionais (estudos na China, EUA, Itália, Canadá, Japão, Coréia do Sul, Suiça, etc) e analisam especificamente pacientes oncológicos. O primeiro estudo focou nos aspectos relacionados ao processo de inserção do PICC (“avaliar intervenções de enfermagem para minimizar a oclusão do cateter PICC”), importante fator de complicações que não é explorado nesse estudo. A segunda referência comparou as complicações entre dois tipos de cateteres (Porta e PICC), especificamente em pacientes oncológicos.

10- A nota de rodapé inserida para explicar “PICC”, na Tabela 3 está assim: ePICC: Cateter Venoso Central de Inserção, falta a palavra periférica. Deve ficar assim: ePICC: Cateter Venoso Central de Inserção Periférica

11- De modo geral, o texto precisa ser revisado para melhorar sua clareza, precisão e objetividade.

12- Informar na folha de rosto de rosto que se obteve dispensa de TCLE. Cabe esclarecer que a falta de contato direto não isenta automaticamente a obtenção de TCLE, pois ainda envolve pesquisa com seres humanos. A dificuldade de obtenção de assinatura do TCLE ou uso de banco de dados antigo ou extenso é que justificaria essa dispensa.

Recommendation: Major revision

